# Microplastic Neurotoxicity in the Prefrontal Cortex: A Review of Mechanisms and Neuropsychiatric Associations

**DOI:** 10.3390/toxics14050359

**Published:** 2026-04-24

**Authors:** Zixuan Liang, Enguo Zhang, Bing Han, Zhenhao Yang, Xiangjing Meng, Yu Zhang, Jiazi Ma, Ziyang Xu, Mengjie Cheng, Hua Shao, Shangya Chen

**Affiliations:** 1 Shandong Academy of Occupational Health and Occupational Medicine, Shandong First Medical University & Shandong Academy of Medical Sciences, Jinan 250062, China; zixxxuan_l@163.com (Z.L.); zenguo@163.com (E.Z.); yh12161208@163.com (Z.Y.); mengxiangjing@email.sdfmu.edu.cn (X.M.); zhangyu@email.sdfmu.edu.cn (Y.Z.); 19560658839@163.com (J.M.); 18085208265@163.com (Z.X.); chengmengjie0713@163.com (M.C.); 2 Department of Head and Neck Surgery, The Third Affiliated Hospital of Shandong First Medical University, Shandong First Medical University & Shandong Academy of Medical Sciences, Jinan 250062, China; hepatobiliary@sina.com

**Keywords:** microplastics, prefrontal cortex, adrenergic receptors, phenylacetylglutamine

## Abstract

The escalating crisis of plastic pollution has positioned microplastics (MPs) as globally pervasive environmental contaminants, with a documented presence across aquatic, terrestrial, and atmospheric ecosystems, as well as within biological organisms. A growing body of evidence suggests that MPs not only threaten ecological integrity but may also induce multifaceted neurotoxic effects in humans, particularly targeting the functional architecture of the prefrontal cortex (PFC). As the central regulator of cognition, emotional processing, and behavioral control, PFC dysfunction has been hypothesized to be associated with cognitive deficits, emotional dysregulation, and behavioral abnormalities. In this comprehensive review, we synthesize the current understanding of MP-mediated neurotoxicity through three interconnected pathways: (1) structural and functional impairment of PFC neural networks, (2) disruption of neurotransmitter homeostasis, and (3) potential associations with neuropsychiatric pathogenesis. By integrating these mechanistic insights, this work aims to provide a scientific foundation for risk assessment frameworks and evidence-based environmental health policies.

## 1. Introduction

Microplastics (MPs), defined as plastic fragments or fibers less than 5 mm in diameter [1], originate primarily from the fragmentation of larger plastic debris (e.g., bags and bottles) and the direct release of synthetic microbeads from commercial products such as cosmetics and personal care formulations. Their persistence due to resistance to biodegradation makes them ubiquitous environmental pollutants. In biological systems, the distribution of these particles is closely associated with their size: micron-scale particles primarily accumulate in tissues such as the gastrointestinal tract; in comparison, nanoscale particles are capable of crossing multiple biological barriers—including cell membranes and the blood–brain barrier (BBB)—entering the systemic circulation and subsequently reaching deeper organs such as the liver, spleen, and even brain tissue [2,3]. Human exposure occurs through multiple routes [4], including dietary intake (via contaminated seafood, drinking water, and processed foods), inhalation (of airborne particles and fibers), and dermal contact (with synthetic textiles and personal care products) [5]. Evidence indicates that MPs can cross critical biological barriers, such as the intestinal epithelium and the BBB, leading to accumulation in various tissues (e.g., liver, kidney, gut, brain) [6,7,8]. Significantly, MPs act as vectors for environmental contaminants, adsorbing and concentrating pollutants such as heavy metals, plasticizers, and persistent organic pollutants (POPs). These co-adsorbed toxicants can subsequently desorb within biological systems, creating a complex co-exposure scenario that elicits a cascade of adverse effects—including oxidative stress, inflammatory responses, and cellular damage [9]. These factors have raised significant concerns about their potential to induce neurotoxic effects in the central nervous system (CNS). While the understanding of microplastic (MP) health impacts remains an evolving field of research, accumulating evidence derived from mammalian models demonstrates that MPs/NPs can induce behavioral abnormalities, cognitive impairments, and neuroinflammation [10,11]. The neurotoxic effects of MPs are primarily mediated through direct physical damage, their role as vectors for adsorbed toxicants, and the inherent chemical toxicity of these co-transported pollutants. These mechanisms act synergistically, ultimately contributing to complex and multifaceted pathological pathways affecting the nervous system [12].

Several recent reviews have addressed the general neurotoxic potential of MPs/NPs [10,11,13,14]. However, none have specifically focused on the PFC as a primary target, nor have they systematically integrated the mechanistic pathways linking MP/NP exposure to PFC-specific structural, neurochemical, and behavioral outcomes. The present review fills this gap by (i) synthesizing evidence for preferential MP/NP accumulation in the PFC; (ii) delineating region-specific mechanisms including neurotransmitter dysregulation (dopamine, GABA/glutamate, and norepinephrine) and glial activation; and (iii) critically evaluating the translational relevance of animal models to human neuropsychiatric risk. This PFC-centered framework provides a novel contribution distinct from prior general neurotoxicity reviews.

## 2. Methodology

In this narrative review, we employed a structured search and selection process to ensure comprehensive literature coverage. A comprehensive literature search was conducted across PubMed, Web of Science, and Scopus using the following key term combination: (“microplastic” OR “nanoplastic” OR “MPs/NPs”) AND (“prefrontal cortex” OR “PFC” OR “executive function”) AND (“neurotoxicity” OR “neuroinflammation” OR “cognition” OR “behavior”).

The vast majority of the literature is concentrated after 2015, indicating that research on microplastics and human health has experienced explosive growth over the past decade. The inclusion criteria comprised the following: (1) Currently published literature focused on MPs/NPs affecting the PFC or its functions [8]; (2) studies reporting molecular, cellular, behavioral, or neuropsychiatric outcomes; and (3) peer-reviewed original articles, reviews, and short communications. Exclusion criteria included the following: (1) studies of other brain regions without PFC data; (2) MP (>5 mm) research without an M/NP component; and (3) conference abstracts and unpublished data.

From 187 initially identified records, 80 studies were included after title/abstract and full-text screening. Findings are categorized by study type (human, animal/in vivo, and in vitro) to clarify the evidence base. A summary table (Table 1) is employed to synthesize key studies, detailing particle characteristics, exposure routes, mechanisms, PFC-related effects, and behavioral outcomes.

## 3. Micro/Nanoplastic (MP/NP) Neurotoxicity

### 3.1. Primary Exposure Routes and Internalization

MPs/NPs primarily enter the human body through three pathways: dietary intake, inhalation, and dermal contact. Among these pathways, ingestion followed by gastrointestinal absorption is the main exposure route [2]. Following internalization, MPs/NPs can accumulate within biological tissues and are subsequently translocated to distant organs via the bloodstream [6]. Their nanoscale dimensions grant NPs a significantly enhanced capacity for tissue penetration. Similarly, MPs with diameters less than 10 μm exhibit greater translocation potential [28].

### 3.2. Toxic Mechanisms and Exposure Pathways

Airborne MPs/NPs inhaled during respiration may deposit in the lungs [29]. While NPs can reach deep alveolar regions and potentially enter systemic circulation [7], larger MPs primarily deposit in the upper respiratory tract [30]. Ingested particles can translocate across the intestinal epithelium [31]. Particles derived from synthetic textiles, which are often fibrous, may also enter via dermal contact [32]. Within biological systems, MPs/NPs can inflict direct physical damage, including frictional abrasion and tissue penetration, particularly associated with fibrous or irregularly shaped particles. Critically, their role as vectors for environmental toxicants is of paramount importance. MPs/NPs efficiently adsorb POPs (e.g., PAHs, dioxins) [33], heavy metals (e.g., cadmium, lead), and plastic additives (e.g., phthalates, bisphenol A). These co-adsorbed contaminants can subsequently desorb within the gastrointestinal tract, local cellular microenvironments, or target organs such as the brain [33], resulting in co-exposure toxicity, also referred to as the Trojan horse effect [34]. The physicochemical properties of MPs/NPs—including particle size, shape, surface charge, and contaminant burden—critically influence their biodistribution, cellular uptake, and ultimate toxicological outcomes [27,35]. Collectively, these properties represent a principal determinant of MP/NP-induced neurotoxicity.

### 3.3. Mechanisms of Neurotoxicity

Exposure to MPs/NPs can induce neurotoxic effects through several interconnected cellular and molecular pathways, with the intensity and specific manifestations modulated by particle physicochemical properties (e.g., size, shape, polymer type, and contaminant load) [13]. This section provides a concise overview of these primary mechanisms. Detailed discussions on how these mechanisms manifest specifically within the PFC and contribute to its structural and functional alterations are presented in Section IV.

**Figure 1:**
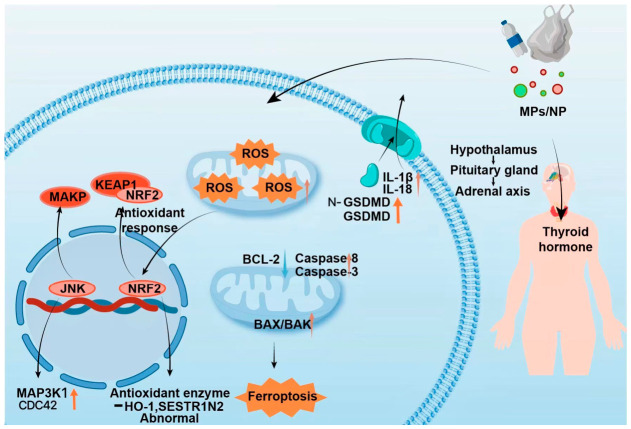
Schematic illustration of the mechanisms underlying microplastic/nanoplastic (MP/NP)-induced toxicity. Exposure to MPs/NPs induces mitochondrial ROS generation, thereby activating the MAPK signaling pathway (including JNK, MAP3K1, and Cdc42) and disrupting the NRF2–KEAP1-mediated antioxidant defense system, as evidenced by aberrant expression of enzymes such as HO-1 and SESTRIN2. Concurrently, MPs/NPs promote apoptotic cell death via downregulation of BCL-2 and upregulation of Caspase-8/-3 and BAX/BAK, in addition to triggering ferroptosis. In addition, they induce pyroptosis-mediated inflammatory responses, characterized by GSDMD cleavage and release of IL-1β and IL-18. Collectively, these cellular disturbances subsequently impair the hypothalamic–pituitary–adrenal axis, ultimately leading to dysregulation of thyroid hormone homeostasis. Note: ↑ indicates up-regulated; ↓ indicates down-regulated.

Oxidative stress is a primary mechanism of MP/NP neurotoxicity, involving excessive reactive oxygen species (ROS) production that damages cellular components [36,37]. This disrupts mitochondrial function, activates stress-response pathways like Nrf2/Keap1, and can lead to cell death. Both in vitro studies confirm that MP/NP exposure increases ROS levels in cerebral cells, which is consistent with evidence from in vivo studies showing elevated oxidative stress in brain tissues [36], with NPs often exhibiting greater potency due to enhanced cellular uptake [34]. This stress (induced by ROS) can suppress the cellular antioxidant response (e.g., via Nrf2/Keap1 dysregulation) [38] and activate pro-apoptotic/inflammatory signals (e.g., via JNK/MAPK) (Figure 1). The neurotoxic impact is frequently exacerbated by co-adsorbed environmental contaminants, such as heavy metals [39].

Neuroinflammation is a key mediator of MP/NP neurotoxicity, often acting in concert with oxidative stress. Exposure triggers microglial activation and upregulates pro-inflammatory cytokines (e.g., TNF-α, IL-1β, and IL-6) [18,40]. NPs may directly enter brain tissue to initiate this response, while larger MPs often act indirectly via systemic inflammation [41]. A central pathway involves the activation of the NLRP3 inflammasome, typically initiated by MP/NP-induced oxidative stress (priming signal) and direct particle effects (activation signal), leading to caspase-1 activation, maturation of IL-1β/IL-18, and pyroptotic cell death [41,42] (Figure 1). MP/NP exposure also activates the NF-κB pathway, contributing to chronic neuroinflammation linked to neurodegeneration [43]. The inflammatory potential is influenced by particle characteristics (e.g., shape) and co-pollutants (e.g., PAHs) [44].

Apoptosis is characterized by mitochondrial dysfunction, cytochrome c release, and altered expression of regulators like BAX, Caspase-3, and BCL-2 [44] (Figure 1). NPs may directly initiate these processes [45], while MPs often do so secondary to inflammation and oxidative stress. Ferroptosis, involving iron-dependent lipid peroxidation, is associated with Nrf2 pathway dysregulation and can be potentiated by metal-laden NPs [45]. Collectively, these processes lead to morphological changes such as dendritic atrophy and synaptic loss, particularly within the PFC [8].

MPs/NPs can disrupt neuroendocrine homeostasis, primarily by interfering with the hypothalamic–pituitary–adrenal (HPA) axis and hormonal signaling (e.g., thyroid and sex hormones) [46] (Figure 1). This interference may alter neuronal excitability and plasticity. NPs, due to their size, may directly affect hypothalamic neurons, whereas MPs often exert indirect effects via systemic pathways [47]. Furthermore, endocrine-disrupting chemicals (EDCs) either released from plastics (e.g., bisphenol A) or co-adsorbed on their surface can mimic or block natural hormones, exacerbating neuroendocrine dysfunction. NPs may be particularly efficient vectors for delivering EDCs to neural targets [48]. This disruption can exacerbate neuroinflammation and oxidative stress, creating a vicious cycle that deteriorates the microenvironment of relevant brain regions [49].

## 4. PFC Structural and Functional Alterations

The PFC, situated in the anterior frontal lobe, mediates higher-order cognitive and executive functions. These functions encompass sensory information integration, planning, impulse inhibition, emotion regulation, sustained attention, and complex decision-making. PFC operations rely on an intricate neural network primarily composed of glutamatergic pyramidal neurons, which provide the main excitatory output, and GABAergic interneurons, essential for local inhibitory control [50]. Optimal PFC function critically depends on the precise regulation of key neurotransmitter systems: norepinephrine (NE) and intrinsic glutamate (Glu) and γ-aminobutyric acid (GABA) signaling. Collectively, these systems maintain the excitation–inhibition (E/I) balance within PFC circuits, which is fundamental for cognitive control, emotional stability, and behavioral regulation [50].

Several converging factors render the PFC uniquely vulnerable to MP/NP-induced damage. A primary determinant is its high baseline metabolic activity and oxygen consumption, which predispose this region to mitochondrial dysfunction and oxidative stress [25]. In addition, the PFC receives dense dopaminergic and noradrenergic inputs from subcortical nuclei, and MP/NP-induced disruption of these neuromodulatory systems directly compromises executive function and emotional regulation [16,51]. Furthermore, the abundance of glutamatergic pyramidal neurons with extensive dendritic arborization renders the PFC exquisitely sensitive to excitotoxicity and dendritic spine loss [8]. Finally, and perhaps most critically, emerging human postmortem evidence indicates that MPs accumulate preferentially in the PFC compared to other brain regions, at concentrations reaching 3345–4917 µg/g [25]. Together, these features distinguish the PFC as a primary target of MP/NP neurotoxicity, setting it apart from other cortical and subcortical areas.

### 4.1. Neuronal Morphological Alterations

Exposure to MPs can induce significant morphological degeneration in PFC neurons, directly compromising synaptic plasticity and neural circuit function [8]. The results of animal studies have revealed that PS-MPs accumulate within PFC tissue following oral administration, with peak accumulation observed at specific doses. This accumulation correlates with pronounced structural damage to PFC neurons, manifested as reduced dendritic complexity and spine density [8], shortened basal dendrites, and decreased dendritic branching points and nodes [8]. These alterations were detectable at an exposure dose as low as 1 mg/kg/day, suggesting high sensitivity of PFC neurons. PS-MP exposure significantly reduces the expression of BDNF, a critical regulator of neuronal survival, dendritic growth, and synaptic plasticity, underscoring the neuromorphotoxic potential of MPs in the PFC. Collectively, morphological alterations such as dendritic atrophy, diminished spine density [8], and BDNF downregulation impair synaptic connectivity and neural information processing within the PFC, thereby establishing a structural basis for cognitive deficits and emotional–behavioral disturbances.

### 4.2. Blood–Brain Barrier Disruption

BBB is a critical physiological structure that protects the brain parenchyma from circulating neurotoxins. Its integrity is essential for maintaining normal PFC function. The results of in vitro studies have shown that microplastics, particularly PS-NPs, are effectively internalized by human brain microvascular endothelial cells (hCMEC/D3). This internalization triggers endothelial damage, manifested through elevated ROS production, activation of the NF-κB signaling pathway, enhanced secretion of tumor necrosis factor-alpha (TNF-α), and the induction of necroptotic cell death [18]. Critically, MP exposure significantly impairs tight junction (TJ) integrity, as evidenced by diminished trans-endothelial electrical resistance (TEER) and downregulation of key TJ-associated proteins (e.g., Claudin-5, Occludin, and ZO-1), concomitant with elevated BBB permeability. In vitro models corroborate the finding that PS-NPs can undergo transcytosis across endothelial monolayers. Correspondingly, the results of in vivo studies in murine models have revealed that MP exposure compromises BBB integrity [43], with smaller particle sizes inducing more pronounced disruption in a dose-dependent manner. BBB disruption not only facilitates the direct translocation of MPs into the PFC parenchyma but also permits the infiltration of peripheral inflammatory mediators and circulating neurotoxins. This cascade exacerbates neuroinflammation and oxidative stress, creating a self-perpetuating cycle that progressively deteriorates the PFC microenvironment.

### 4.3. Glial Cell Activation

**Figure 2:**
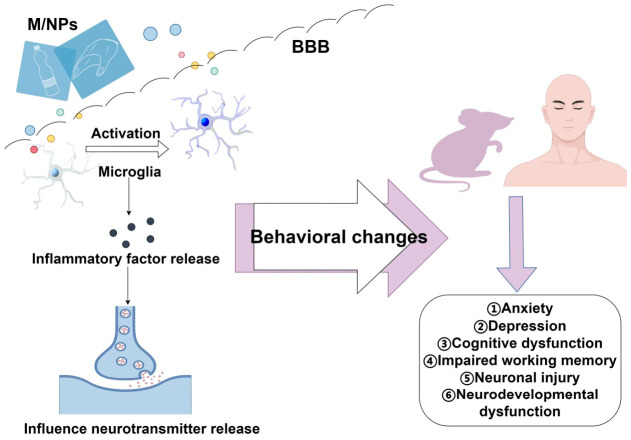
Pathway from M/NP exposure to neurobehavioral impairment via microglial activation. M/NPs cross the BBB → activate microglia → release inflammatory factors → disrupt neurotransmitter release → induce behavioral/neuronal alterations (anxiety, depression, cognitive dysfunction, working memory deficits, neuronal damage, and neurodevelopmental abnormalities) in rodents and humans.

Microglia, the resident immune phagocytes of the central nervous system (CNS), play a pivotal role in mediating the PFC’s response to MP exposure (Figure 2). MPs, such as polystyrene (PS) particles, can directly activate murine microglial cell lines (e.g., BV2) and macrophage cell lines (e.g., RAW264.7) [22]. This activation is evidenced by morphological transformation into an amoeboid phenotype and dose-dependent upregulation and secretion of pro-inflammatory cytokines (e.g., tumor necrosis factor-alpha [TNF-α], interleukin-1 beta [IL-1β], and interleukin-6 [IL-6]) [22]. Notably, microplastic exposure induces pyroptosis in these immune cells, characterized by the upregulation of Gasdermin D (GSDMD)—a key executor protein of pyroptosis—and its cleaved N-terminal fragment (N-GSDMD), with pyroptotic cell death exhibiting a dose-dependent increase [22]. This effect is markedly attenuated by GSDMD silencing. Conditioned medium derived from MP-stimulated BV2 microglia induces significant cytotoxicity in co-cultured murine neuronal cells (e.g., HT-22), indicating that microglia-derived soluble mediators, particularly pro-inflammatory cytokines, constitute a key mechanism through which MPs indirectly impair PFC neurons [22]. Crucially, this mechanism aligns with the established pathological process wherein sustained microglial activation and localized pyroptosis contribute to the establishment of a chronic neuroinflammatory milieu within the PFC [52]. This neurotoxic environment is characterized by the sustained release of cytotoxic substances that inflict direct damage on adjacent neurons and compromise synaptic integrity [53] (Figure 2).

### 4.4. PFC Neurotransmitter System Perturbation

MP exposure has been demonstrated to significantly perturb key neurotransmitter systems within the PFC, including DA, Glu, GABA, and NE pathways. These disruptions are primarily driven by key mechanisms, including oxidative stress, neuroinflammation, mitochondrial dysfunction, and direct interference with neurotransmitter receptors [54]. The disruption of these critical systems thereby impairs the excitatory–inhibitory (E/I) balance within PFC neuronal circuits. This dysregulation is considered a key mechanism underlying the observed cognitive deficits, emotional dysregulation, and associated behavioral abnormalities.

#### 4.4.1. Dopaminergic System Dysregulation

**Figure 3:**
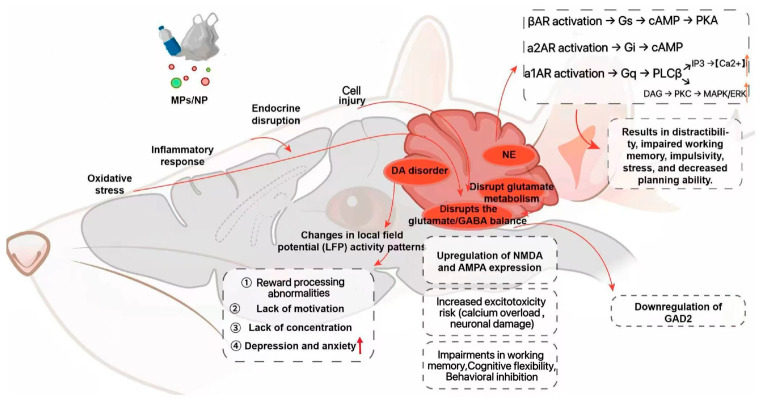
Schematic illustration of the neurotoxic mechanisms induced by MPs/NPs. MPs/NPs initiate neurotoxicity through oxidative stress, inflammatory responses, and endocrine disruption, leading to multifaceted cellular impairments. Key molecular alterations include the following: DA and NE signaling; disruption of glutamate metabolism and glutamate/GABA balance, whereby upregulation of NMDA/AMPA receptors promotes excitotoxicity—characterized by calcium overload and neuronal damage—while downregulation of GAD2 impairs GABA synthesis; and adrenergic receptor activation (βAR, α2AR, and α1AR) engaging G protein-coupled pathways, with these changes corresponding to altered local field potential (LFP) patterns, which are associated with behavioral abnormalities such as motivational deficits, inattention, and anxiety-like phenotypes. Ultimately, these cascading disruptions contribute to cognitive–behavioral deficits, including impaired working memory, increased impulsivity, and reduced stress resilience, illustrating the multi-level neurotoxicity of MPs/NPs. Note: ↑ indicates up-regulated.

The dopaminergic system orchestrates PFC-mediated regulation of executive functions, motivation, and affective states. Accumulating evidence indicates that MP exposure during critical developmental periods alters behavioral responsiveness to dopaminergic agents (e.g., tetrabenazine [TBZ], L-3,4-dihydroxyphenylalanine [L-DOPA]) in murine models, suggestive of underlying dopaminergic dysfunction. Furthermore, MP exposure modulates local field potential (LFP) activity patterns within key brain regions interconnected with the PFC (Figure 3), including the striatum, nucleus accumbens, and amygdala [16]. These modulations are characterized by alterations in spectral power, particularly increased power within specific frequency bands, which may reflect or perturb dopaminergic circuit dynamics. Disrupted DA signaling within the PFC is posited to underlie impairments in reward processing [55], diminished motivation, deficits in impulse control and attentional processes, and heightened susceptibility to anxiety- and depression-like behaviors [55,56].

#### 4.4.2. GABA–Glutamate Dysregulation and E/I Imbalance

Glu serves as the principal excitatory neurotransmitter, whereas GABA functions as the primary inhibitory neurotransmitter within the PFC. The precise maintenance of the E/I balance between these systems is fundamental for synaptic integration and higher-order cognitive processes (Figure 3). MP exposure, through mechanisms such as oxidative stress and mitochondrial dysfunction, may disrupt glutamate metabolism and clearance. This disruption is partially attributable to the impairment of astrocytic function [57]. Specifically, PS-NPs have been shown to downregulate the expression and/or activity of crucial astrocytic glutamate transporters [58], including excitatory amino acid transporter 1 (EAAT1/GLAST) and excitatory amino acid transporter 2 (EAAT2/GLT-1). This downregulation leads to elevated extracellular glutamate concentrations and delayed clearance kinetics in the PFC. Furthermore, modifications in the expression, subunit composition, and functional characteristics of glutamate receptors—including N-methyl-D-aspartate (NMDA) and α-amino-3-hydroxy-5-methyl-4-isoxazolepropionic acid (AMPA) receptors—have been consistently observed. This glutamate dysregulation likely results from direct oxidative stress-induced damage to astrocytes and neurons [59].

Concomitantly, PS-NP exposure has been shown to reduce the expression of glutamic acid decarboxylase 2 (GAD2), the rate-limiting enzyme for GABA synthesis, in the thalamus. Moreover, it significantly decreases GABA concentrations within the PFC and amygdala of offspring mice, particularly following maternal exposure. These GABAergic deficits likely reflect oxidative stress-mediated suppression of GABA synthesis and/or functional impairment of GABAergic neurons [20]. The disruption of these critical systems thereby impairs the excitatory–inhibitory (E/I) balance within PFC neuronal circuits. This dysregulation is considered a key mechanism underlying the observed cognitive deficits, emotional dysregulation, and associated behavioral abnormalities. Excitotoxicity is typified by glutamate-induced calcium dysregulation and subsequent neuronal injury. Collectively, these alterations in the glutamate–GABA systems compromise the stability of cortical neural networks and impair critical functions such as working memory, cognitive flexibility, behavioral inhibition, and emotional regulation [60].

#### 4.4.3. Noradrenergic System Dysfunction

Under the pathological conditions induced by microplastic exposure, noradrenergic system function is also significantly compromised [51]. As microplastics reduce norepinephrine and induce neuroinflammation, they disrupt neurotransmitter balance and worsen neurotoxicity via pro-inflammatory cytokines like IL-6 and IL-1β [51]. Direct evidence for MP/NP-induced disruption of noradrenergic signaling in the PFC is currently limited. However, based on established principles of PFC neurobiology [61,62,63], dysregulation of NE pathways—which can result from MP/NP-induced oxidative stress and neuroinflammation [51]—would be expected to impair PFC function. Specifically, activation of β-adrenergic receptors (β-ARs) enhances synaptic plasticity and neurotransmission through the Gs–cAMP–PKA signaling cascade; dysregulation of this pathway is implicated in aberrant emotional reactivity and heightened stress sensitivity [61]. It should be emphasized that these mechanisms are inferred from general neurobiology rather than directly demonstrated in MP/NP-exposed animals. Future studies directly measuring NE signaling in the PFC following MP/NP exposure are needed to validate this pathway. Conversely, the activation of α2A-AR is crucial for maintaining working memory, with its mechanism lying in the suppression of neuronal overexcitability [62,63]. This is evidenced by improved primate dlPFC performance with α2-AR agonists [62] and high receptor expression in cognitive brain regions [63]. Perturbations of these NE signaling pathways can significantly impair cognitive control functions (Figure 3). This dual dysregulation—featuring glutamatergic hyperexcitation (from impaired clearance and altered receptor function) combined with GABAergic hypo-inhibition (from reduced enzyme expression and neurotransmitter availability)—thus elevates the risk of excitotoxicity.

## 5. Induced PFC Injury and Behavioral Pathological Outcomes

MPs, particularly nanoscale particles, can traverse the BBB and accumulate within the PFC, inducing key neuropathological alterations, including neuroinflammation, oxidative stress, synaptic dysfunction, and neuronal apoptosis. These PFC-specific pathologies underlie the manifestation of distinct behavioral phenotypes, encompassing cognitive impairments (e.g., deficits in learning and memory), emotional dysregulation (e.g., anxiety- and depressive-like behaviors), and aberrant social interactions. Critically, prolonged exposure to these particles and subsequent impairment of the PFC may elevate the risk of neurodevelopmental disorders—such as autism spectrum disorder (ASD) and attention-deficit/hyperactivity disorder (ADHD)—in addition to neurodegenerative diseases, including Alzheimer’s disease (AD). In a case–control study, the median microplastic concentration in the frontal cortex of dementia patients was 26,076 µg g^−1^—approximately five-fold higher than that in age-matched controls. After multivariate adjustment, this difference remained statistically significant, supporting an association between elevated microplastic accumulation in this brain region and cognitive impairment/dementia [50].

### 5.1. Prefrontal Cortex Damage and Cognitive Dysfunction

As a principal cognitive region, the brain mediates MP-induced deficits. Behavioral tests (Morris water maze, novel object recognition, and Y-maze) show MPs impair learning/memory, indicated by prolonged latencies, poor novel object discrimination, increased hyperactivity, and altered spontaneous alternation [21,23]. These deficits correlate with disrupted neurotransmitter gene expression and suppressed GABAergic signaling with reduced synaptic proteins (e.g., PSD95) [21]. While direct evidence for MP-induced disruption of cortical excitatory-inhibitory balance in the PFC remains to be established, it is plausible—based on established neurobiological principles and findings from analogous neurotoxicity models (e.g., THC-induced GABAergic disinhibition [64])—that suppressed GABAergic signaling triggered by MPs may similarly perturb this balance, thereby contributing to memory impairment. These PFC-linked cognitive impairments are mechanistically associated with dysregulated neurotransmitter systems (particularly DA and NE pathways) within the PFC [65].

### 5.2. Prefrontal Cortex Damage-Mediated Emotional Dysregulation

PFC is critically involved in emotional regulation and stress response modulation, and its dysfunction constitutes a key pathophysiological mechanism underlying environmental microplastic-induced affective disturbances [66,67].

#### 5.2.1. Anxiety-like Phenotypes

MP exposure induces pronounced anxiety-like behaviors in rodent models. In the open field test (OFT), exposed subjects exhibit significantly reduced central zone exploration time and fewer central entries [19]. During elevated plus maze (EPM) assessments, these animals demonstrate markedly decreased open arm time and reduced open arm entries [24]. Consistent with gut–brain axis mechanisms (e.g., gut microbiota affecting PFC function in depressive disorder [68]), MP-induced prefrontal dysfunction-related behavioral changes may also involve gut microbiota dysbiosis and systemic inflammation. Furthermore, localized neuroinflammation within the brain—characterized by HRAS-mediated PERK–NF-κB pathway activation, microglial hyperactivation [69], and pro-inflammatory cytokine release—coupled with neurotransmitter imbalances (e.g., involving serotonin [5-HT], GABA, and NE) collectively drive the anxiety phenotype [70].

#### 5.2.2. Depression-like Phenotypes

Chronic exposure to polystyrene nanoplastics (PS NPs) has been associated with depression-like behaviors in adolescent C57BL/6J mice, manifested as increased immobility and reduced struggling in the forced swim test (FST) and tail suspension test (TST), without locomotor impairment [19]. A proposed mechanism involves overactivation of astrocytes and downregulation of the glutamate transporter EAAT2 in the medial prefrontal cortex (mPFC), and the EAAT2 activator LDN-212320 has been shown to ameliorate these behavioral and synaptic deficits [19]. Furthermore, PS NP exposure has been linked to mitochondrial dysfunction, neuronal apoptosis, localized neuroinflammation, and downregulated monoaminergic neurotransmission in the mPFC [26]. It also has been associated with abnormal expression of lncRNAs, miRNAs, and mRNAs, which may disrupt key pathways such as axon guidance through multiple regulatory modes [71]. These pathological alterations have been suggested to be associated with the accumulation of PS NPs and potential functional impairment in the mPFC.

### 5.3. Prefrontal Cortex Damage and Social Deficits

As the hub for social cognition, the PFC underpins social function. Long-term MP exposure in rodents induces social deficits: mice exposed to larger polystyrene particles (≥100 nm) show reduced social interest, linked to dysregulated PFC neurotransmitter genes (e.g., Grm5, Adora2a) [21]. This exposure also impairs learning/memory and increases anxiety [21,23], both tied to social dysfunction. The disorders relate to these mechanisms: These behavioral impairments are mechanistically associated with the following:

(i) MP-induced neuronal damage in PFC subregions critical for social information processing (e.g., medial prefrontal cortex [mPFC]);
(ii) Dysregulation of key neuromodulatory systems, including oxytocinergic [OT] and DA pathways;
(iii) Sustained neuroinflammatory responses within the PFC.

### 5.4. Developmental PFC Disruption and Neurodevelopmental Disorders

The developing PFC exhibits heightened vulnerability to exposure due to BBB immaturity, potentially underlying increased susceptibility to neurodevelopmental disorders [14]. Prenatal or early postnatal exposure induces persistent behavioral phenotypes in offspring [26], including the following:

(i) Hyperactivity and attentional deficits (ADHD-like phenotypes);
(ii) Impaired social interaction (reflecting ASD-associated traits);
(iii) Cognitive impairments;
(iv) Aberrant anxiety responses [72].

Notably, sustained low-dose exposure (10 μg/kg/day) to 23 nm polystyrene nanoplastics induces hyperactivity-like phenotypes and increased risk-taking behavior in mice; in contrast, exposure to plastic particles with larger sizes (≥40 nm) may significantly suppress the mice’s locomotor activity, showing size-dependent differences in behavioral effects [21,24]. Preclinical evidence suggests a potential link between developmental exposure and increased susceptibility to ASD and ADHD, although direct clinical data are currently lacking [73]. Mechanistically, M/NPs disrupt critical neurodevelopmental processes within cerebral cortex, including neurogenesis, neuronal migration, synaptogenesis, and myelination [74]. These particles further induce oxidative stress, sustained neuroinflammation, and endocrine disruption—such as thyroid hormone dysregulation and sex steroid imbalance [75,76]—ultimately disrupting normal prefrontal cortical development and increasing susceptibility to ASD, ADHD, and intellectual disability (ID). For example, researchers demonstrated that maternal exposure to 50 nm PS-NPs (1 mg/kg) during gestation resulted in ADHD-like behaviors (hyperactivity and inattention) in offspring mice, accompanied by reduced PFC DA concentration and impaired synaptic plasticity [21].

### 5.5. Cumulative PFC Damage and Neurodegeneration

Chronic M/NP exposure induces sustained injury to the PFC, characterized by progressive neuronal loss, synaptic dysfunction, chronic neuroinflammation, mitochondrial impairment, and disruption of proteostasis. These pathological alterations collectively constitute a core framework underpinning neurodegeneration. In AD mouse models, administration of PS-MPs (2.5 μm, 10 mg/kg/day) exacerbated PFC-associated cognitive deficits. Mechanistically, researchers observed a significant increase in the N-GSDMD/GSDMD ratio, confirming enhanced microglial activation and pyroptosis, combined with elevated expression of local pro-inflammatory cytokines (IL-1β, IL-6, and TNF-α) [22]. The results of mechanistic studies indicate that MPs elicit oxidative stress in cortical neurons, leading to NMDA and IP3 receptor-mediated intracellular Ca^2+^ overload, thereby triggering both apoptotic and autophagic pathways [77]. Activated microglia further exacerbate neuronal damage in the frontal cortex by continuously releasing pro-inflammatory mediators and activating downstream signaling pathways, thereby promoting the neurodegenerative progression of AD [78]. Cumulatively, sustained neuronal loss, glutamatergic signaling dysfunction, chronic neuroinflammation, mitochondrial dysfunction, and proteostasis disruption—including the potential facilitation of Aβ aggregation and tau hyperphosphorylation—represent key mechanistic links connecting exposure to the pathogenesis of neurodegenerative disorders [79]. A recent study reported greater accumulation of M/NPs in decedent human brains with documented dementia diagnoses, with notable deposition in cerebrovascular walls and immune cells [25]. These findings highlight the need for further research to elucidate the potential role of M/NPs in neurodegenerative disease progression.

## 6. Summary

In this review, we comprehensively synthesize current research on the neurotoxic effects of MPs on the PFC. Substantial evidence indicates that MPs can enter the human body through multiple routes of exposure. Notably, nanoparticle-sized MPs are capable of not only crossing the BBB but also accumulating preferentially in the PFC. Within the PFC, MPs initiate a cascade of interconnected pathological processes, mediated both by their inherent physical properties and their role as vectors for chemical pollutants, encompassing oxidative stress (characterized by excessive ROS generation and compromised antioxidant defenses); neuroinflammation (manifesting as glial activation, pro-inflammatory cytokine release, and pyroptosis); activation of cell death pathways (including apoptosis, pyroptosis, ferroptosis, and dysregulated autophagy); BBB disruption (involving endothelial damage, tight junction impairment, and increased permeability); neurotransmitter system dysfunction (affecting DA, Glu/GABA, and NE signaling); neuroendocrine disturbances (e.g., dysregulation of the HPA axis, thyroid hormones, and sex hormones); and perturbations along the gut–brain axis (primarily via intestinal barrier compromise and gut microbiota dysbiosis).

**Figure 4:**
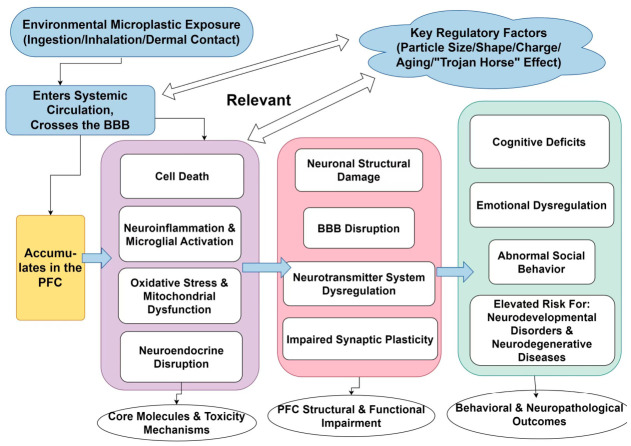
Mechanisms of environmental microplastic (MP) and nanoplastic (NP) exposure-induced neurotoxicity and behavioral pathology in the PFC. Dashed lines indicate pathways primarily associated with NPs (due to enhanced barrier penetration), while solid lines indicate pathways relevant to both MPs and NPs.

These pathophysiological processes act synergistically, culminating in dendritic atrophy, reduced spine density, downregulation of BDNF, impaired synaptic plasticity, diminished neuronal connectivity, and aberrant electrophysiological activity within PFC neurons. As a result, PFC dysfunction presents as significant cognitive deficits, affective and behavioral alterations (e.g., anxiety and depression), reduced social interaction, and an increased risk of neurodevelopmental (e.g., ASD and ADHD) and neurodegenerative disorders (e.g., AD). Further details are provided in Figure 4.

## 7. Limitations and Future Perspectives

### 7.1. Key Limitations of Current Research

Despite significant progress in understanding mediated PFC neurotoxicity, several critical limitations remain:

Dose and Exposure Relevance: Many experimental studies involve the use of high, non-environmentally relevant doses that exceed typical human exposure levels. The neurotoxic effects of chronic, low-dose exposure to environmentally relevant concentrations remain poorly characterized. A critical limitation of the current evidence base is the widespread use of exposure doses that vastly exceed environmentally relevant levels. While human dietary and inhalation exposure to MPs/NPs is estimated in the range of micrograms to low milligrams per day [80], many rodent studies employ daily doses of 10–100 mg/kg [19,22]—several orders of magnitude higher. Furthermore, intravenous injection [22] and intratracheal instillation [77] do not reflect typical human exposure routes, which primarily involve ingestion of contaminated food/water and inhalation of airborne particles. Although such high-dose paradigms are useful for mechanistic proof-of-concept and hazard identification, their direct extrapolation to human risk assessment is problematic. Chronic low-dose protocols using environmentally relevant concentrations (e.g., 0.1–1000 μg/L in drinking water [23]) and physiologically realistic exposure routes should be prioritized in future studies to improve translational relevance.

Translational Gaps: Current evidence is predominantly derived from animal models (rodents and zebrafish) and in vitro systems [13], with limited direct clinical/epidemiological data in humans. Significant differences in physiology, metabolism, and BBB structure between animal models and humans may limit the translational relevance of findings. Moreover, rodent behavioral assays (e.g., open field test, elevated plus maze) capture only limited dimensions of human neuropsychiatric conditions and should be viewed as behavioral analogs rather than direct clinical equivalents. Observed anxiety-like or social deficits in animal models do not necessarily imply causation in humans. Notably, a recent postmortem study [25] provides direct evidence of MP accumulation in the human PFC, with concentrations in dementia cases approximately five-fold higher than those in age-matched controls. However, this cross-sectional association cannot establish causality (e.g., reverse causation or residual confounding cannot be excluded), and such direct human evidence is currently limited to this single study. Direct longitudinal evidence for MP-induced developmental neurotoxicity or neurodegeneration in humans remains lacking.

Heterogeneity of Evidence and Dose–Response Paradoxes: Inconsistencies exist across studies, including the apparent paradox whereby the results of some studies show significant effects at low doses while others require high exposures. Potential factors contributing to this heterogeneity include particle characteristics (size, polymer type, surface charge, and morphology), exposure duration, model system differences, and the possibility of non-monotonic dose–response relationships.

Mixed Exposure Complexity: In the environment, they commonly co-occur with adsorbed chemical contaminants (e.g., heavy metals and POPs) and other pollutants [13]. The synergistic, additive, or antagonistic neurotoxic interactions resulting from such complex “mixed exposures” on PFC function have not been adequately elucidated.

PFC Preferential Accumulation Evidence: While the results of some studies suggest brain region-specific accumulation of MPs, direct comparative evidence for PFC preferential targeting remains limited. The mechanisms underlying potential PFC-specific accumulation (e.g., region-specific receptors and blood flow patterns) require further investigation [81].

Neuropsychiatric Association Evidence: Current associations between exposure and neurodevelopmental/neurodegenerative disorders (ASD, ADHD, and AD) are primarily based on mechanistic preclinical data and theoretical parallels, with a lack of direct clinical evidence establishing causal relationships [82].

### 7.2. Future Research Directions

To address these limitations and drive progress in the field, the authors of future studies should prioritize the following areas:

Mechanistic Elucidation:

Identify the factors governing the selective accumulation of MPs within the PFC, including region-specific BBB permeability and receptor-mediated uptake mechanisms.

Delineate the molecular mechanisms underpinning PFC damage, including the involvement of specific receptors, ion channels, and epigenetic modifications.

Systematically characterize the spatiotemporal dynamics of key dysregulated signaling pathways, including the Nrf2, NF-κB, JNK/MAPK, and PINK1/Parkin pathways, in addition to those downstream of adrenergic receptors.

Exposure Realism:

Conduct long-term studies using environmentally relevant doses and exposure routes (e.g., dietary and inhalation) to better mimic human exposure scenarios.

Investigate the cumulative neurotoxicity of chronic low-dose exposure and potential synergistic effects of mixed-contaminant exposures.

Translational Research:

Develop sensitive and specific early-stage biomarkers (e.g., molecular markers in blood/cerebrospinal fluid and neuroimaging signatures) for induced PFC injury.

Conduct rigorous, large-scale epidemiological studies integrating comprehensive environmental exposure assessment, biological sample analysis (MP quantification and biomarker measurement), and standardized neuropsychological evaluations to establish robust causal relationships between exposure and PFC-associated neuropsychiatric outcomes.

Therapeutic and Preventive Strategies:

Develop mechanism-targeted therapeutic interventions (e.g., antioxidant agents, anti-inflammatory compounds, pyroptosis/apoptosis inhibitors, and BBB protectants) to mitigate M/NP-induced PFC damage.

Explore microbiota-targeted approaches (e.g., probiotics) to modulate the gut–brain axis and reduce neuroinflammatory responses.

Methodological Advances:

Leverage advanced technologies such as spatial transcriptomics, PFC-specific genetic manipulation models (e.g., CaMKIIα-Cre), and in vivo imaging to directly delineate PFC-specific pathological alterations.

Standardize exposure models and analytical methods for detection in biological tissues to improve reproducibility across studies.

### 7.3. Translational Implications and Research Gaps

The translational potential of neurotoxicity research lies in its ability to inform environmental health policies and risk assessment frameworks. Key translational implications include the following:

Biomarker Development: Combining detection in biological fluids (blood) with inflammatory/neurodegenerative biomarkers (e.g., IL-1β, TNF-α, BDNF, and tau) could enable early identification of individuals at risk of PFC damage [83].

Regulatory Policies: Environmental monitoring of MPs in food, water, and air should be strengthened to establish safe exposure limits, particularly for vulnerable populations (children, pregnant women) [80,84].

Public Health Interventions: Awareness campaigns regarding plastic pollution and its potential health impacts could reduce human exposure. Development of biodegradable alternatives to conventional plastics may mitigate long-term environmental accumulation [85].

Clinical Practice: Clinicians should consider exposure as a potential environmental risk factor for cognitive and emotional disorders, particularly in patients with no known genetic or lifestyle risk factors [86].

Critical research gaps that need to be addressed to enhance translational impact include the following:

(1) Establishing causal relationships between exposure and human neuropsychiatric outcomes;
(2) Identifying threshold exposure levels for PFC damage;
(3) Developing effective interventions to reduce exposure and mitigate neurotoxic effects;
(4) Integrating exposure assessment into routine environmental health monitoring.

## 8. Conclusions

In summary, mounting evidence from preclinical studies suggests that MPs may induce PFC neurotoxicity via oxidative stress, neuroinflammation, and synaptic dysfunction, yet critical translational gaps remain. Direct causal evidence in humans is currently limited, and findings should be interpreted with appropriate caution. Future efforts must prioritize human-relevant exposure studies, the development of early diagnostic biomarkers, and the establishment of causal links to neuropsychiatric disorders. Pending such validation, these insights may ultimately be informative. These insights should directly inform public health policies—such as setting safety limits and enhancing environmental surveillance—while raising clinical awareness of MPs as a potential modifiable neurological risk factor. Ultimately, addressing these challenges is essential to mitigate the growing global burden of plastic pollution on brain health.

## Figures and Tables

**Table 1:** Summary of key studies on microplastic-mediated PFC neurotoxicity.

Particle Category	Model/Exposure Conditions	Key Mechanisms	Major Findings
NPs (20–100 nm)	Pregnant SD rats (intratracheal) [7]; pregnant C57BL mice (oral) [15]; male ICR mice (oral) [16]; male BALB/c mice (oral) [17]; male C57BL/6J mice (oral) [18,19]; pregnant mice (oral) [20]; male mice (oral, lifelong) [21]	Placental–fetal translocation; BBB disruption; ROS/apoptosis; GABA disorder; dopamine dysregulation; astrocyte activation; EAAT2 downregulation; mitochondrial dysfunction; microglial activation; necroptosis; neural stem cell disruption; synaptic impairment	NPs cross the placenta into the fetal brain/PFC; PFC/cortex accumulation; mPFC damage with spines/synaptic transmission ↓; GABA/GAD2; LFP increase/circuit abnormality ↓; tight junction injury; neuron damage; abnormal brain development in offspring. * Fetal/placental weight loss; offspring anxiety and defects; locomotion ↓; sociality ↓; anxiety/depression-like behaviors; ADHD-like phenotype; impaired brain aging; EAAT2 activator rescues deficits. ^#^
MPs (500 nm–20 μm)	Male Swiss mice (oral) [8]; APP/PS1 AD mice (i.v.) [22]; male BALB/c mice (drinking water) [23]; adult mice (oral) [24]	BDNF downregulation; neuronal damage; microglial pyroptosis; neuroinflammation; BBB damage; synaptic disorder; neurotransmitter imbalance; gut dysbiosis	PFC accumulation; Nissl bodies/spines/BDNF ↓; inflammation ↑; aggravated AD pathology; spine density ↓; gut–brain axis disruption. * Severe learning/memory impairment (AD model); learning/memory deficit (180d drinking water); anxiety-like behaviors. ^#^
Mixed MNPs (1 nm–5 mm)	Human postmortem frontal cortex [25]; human + rodents (inhalation/ingestion) [26]	Brain accumulation; BBB penetration; oxidative stress; Aβ aggregation; inflammation	High PFC enrichment of nanoscale shards; cross BBB; frontal cortex accumulation. * MNP ↑ in dementia cases; cognitive decline link; dementia risk ↑. ^#^
PS-NPs (inhaled)	Mice; inhalation [27]	Brain deposition; neuronal alteration	NPs deposit in the brain. * Altered animal behaviors. ^#^

* Microscopic findings: neuropathological and biochemical effects in the PFC; ^#^ macroscopic outcomes: behavioral and cognitive outcomes. Note: ↑ indicates up-regulated; ↓ indicates down-regulated.

## Data Availability

No new data were created or analyzed in this study. Data sharing is not applicable to this article.
